# E-Cadherin Immunostaining in Equine Melanocytic Tumors

**DOI:** 10.3390/ani13132216

**Published:** 2023-07-06

**Authors:** José Pimenta, Isabel Pires, Justina Prada, Mário Cotovio

**Affiliations:** 1 Veterinary Sciences Department, University of Trás-os-Montes e Alto Douro, 5000-801 Vila Real, Portugal; ipires@utad.pt (I.P.); jprada@utad.pt (J.P.); mcotovio@utad.pt (M.C.); 2 CECAV—Veterinary and Animal Research Center, University of Trás-os-Montes e Alto Douro, 5000-801 Vila Real, Portugal; 3 Associate Laboratory for Animal and Veterinary Sciences (AL4AnimalS), 5000-801 Vila Real, Portugal

**Keywords:** equine, melanocytic tumors, E-cadherin

## Abstract

**Simple Summary:**

In comparison with other species, a large knowledge gap remains in Equine melanocytic tumors regarding their uncommon benign behavior, since invasion and metastasis are rarely present. Melanocytic tumors invasion and metastization have been associated with Epithelial-Mesenchymal Transition, where the disruption of cell-adhesion molecules has a crucial role. E-cadherin is one of the most prominent adhesion molecules, and the loss of its expression is observed in malignant tumors being associated with aggressive behavior. This study aimed to evaluate E-cadherin immunostaining in equine melanocytic tumors. There was high immunolabeling of E-cadherin in most tumors, with 70.7% of melanomas remaining with high immunostaining. The typical loss of immunostaining in malignant tumors was not observed, and there were no differences between malignant and benign tumors. The high E-cadherin expression is well correlated with the benign biological behavior of equine melanocytic tumors being in accordance with the genetic development factors associated with this neoplastic disease in horses.

**Abstract:**

Melanocytic tumors are an important neoplastic disease in human and veterinary medicine, presenting large differences regarding tumor behavior between species. In horses, these tumors present a prolonged benign behavior, with rare invasiveness and metastases. In humans and small animals, invasion and metastasis have been associated with an Epithelial-Mesenchymal Transition, where the loss of E-cadherin expression plays a key role in tumor progression. This process and the role of E-cadherin have not yet been evaluated in equine melanocytic tumors. This study aimed to assess the immunolabeling of E-cadherin in equine melanocytic tumors and relate this with clinicopathological variables. A total of 72 equine melanocytic tumors were classified as benign and malignant and evaluated by immunohistochemistry for E-cadherin expression. A different pattern of immunostaining was found, contrasting with other species. A total of 69.4% of tumors presented raised immunolabeling of E-cadherin, with 70.7% of melanomas remaining with high expression. The typical loss of immunostaining was not seen in malignant melanomas and no differences were found between benign and malignant melanomas regarding E-cadherin immunostaining. The high immunolabeling of E-cadherin may contribute to the low invasiveness of these tumors, and it is in accordance with the benign behavior of equine melanoma and with the genetic factors associated with its development.

## 1. Introduction

Melanocytic tumors are a devastating disease, frequently diagnosed in multiple species including humans, dogs, and horses. However, there are many differences in clinical and pathological features as well as in the biological behavior of melanocytic tumors between these species [1,2,3].

In humans, the cutaneous melanocytic tumor is the most common type with malignant forms (melanoma) presenting very aggressive behavior with high invasiveness and metastatic rates, conducting to death in most cases [4]. In dogs, both cutaneous and oral melanocytic neoplasms are common, however, they present different biological behavior. Oral melanomas are characterized for being fast-growing tumors, very invasive and metastasizing quickly and frequently to regional lymph nodes and distant organs. In contrast, cutaneous melanomas in dogs are usually benign, with the exception of mucocutaneous that are often malignant and more aggressive. Regardless, they all appear to be, or become with time, somewhat more aggressive in dogs than in horses [3]. Although similar in clinical behavior, these two species (human and dog) differ in terms of etiological and risk factors for this neoplastic disease [1]. Of the three mentioned species, equine melanocytic tumors are the ones with the most atypical behavior. These tumors frequently involve the skin and mucocutaneous regions such as the perianal area, ventral tail surface, eyelids, and lips [5,6]. Etiologically they have a hereditary cause related to an autosomal dominant genetic trait that confers grey coat color (Sintaxin_17_ gene), in contrast with other species where the disease develops through acquired somatic mutations [2,7,8]. Regarding biological behavior, equine melanocytic tumors present an extended period of benign behavior characterized by more or less pronounced mass growth but without evidence of deep invasion. Furthermore, clinically evident metastization is quite rare. However, although clinically benign, these tumors can cause tremendous health concerns due to the direct or indirect impact of mass growth and, with time, malignant transformation of previous benign tumors can occur. Intriguingly, its benign behavior continues in most cases, even after histological features of malignancy have been acquired [9,10,11,12,13].

In a state of homeostasis, melanocyte growth is controlled by keratinocytes through paracrine factors and cell-to-cell adhesion molecules. Dysregulation of this controlling system can lead to uncontrolled proliferation of melanocytes leading to melanocytic tumor formation [14,15,16]. Furthermore, after a phase of tumor growth, tumor cells can start to invade deeper tissues and spread to other locations. For this to happen, tumor cells need to detach from the primary tumor in a process called Epithelial-Mesenchymal Transition (EMT), a process by which epithelial cells lose their adhesions, acquire a mesenchymal shape and become mobile. Although melanocytic tumors are not epithelial in origin, an EMT-like process does occur, where these tumors acquire a more aggressive behavior, being well described in human medicine literature. One of the main initial steps of this process is the dysregulation of cadherins, namely the epithelial cadherin (E-cadherin) [16,17].

Cadherins are cell-adhesion calcium-dependent proteins that play an important role in tissue integrity, organization, and development. E-cadherin is expressed in melanocytes and keratinocytes participating in the connection between them and allowing this way the regulation of melanocytes growth and proliferation. During malignant transformation there is a loss of E-cadherin expression which has an important role in the EMT-like process, allowing the melanoma to become invasive a metastasize [14,15,18,19].

E-cadherin studies in veterinary medicine focus mainly on epithelial tumors, with the literature on melanocytic tumors being scarce [1]. The few reports about its expression are demonstrated in dogs, presenting similar finding to human melanocytic tumors, were a downregulation of E-cadherin was found in malignant melanomas comparing with benign melanomas [20,21,22,23,24].

The aim of this work was to study E-cadherin immunostaining in equine melanocytic tumors and evaluate differences between benign and malignant melanomas in order to possibly correlate its immunolabeling with the biological behavior of these tumors in horses. We also try to evaluate possible correlations with clinicopathological features.

## 2. Materials and Methods

### 2.1. Tissue Samples

Formalin fixed paraffin-embedded tissues samples of primary cutaneous or mucocutaneous equine melanocytic tumors, with a previous histological diagnosis, were included in this retrospective study.

### 2.2. Clinical Information

The available clinical reports were collected, and the following information was recorded: age, gender, breed, coat color, and mass localization. Not all cases had the complete clinical information available. Regarding age, three categories were created: young: ≤5 years old; adult: between 6 and 14 years; geriatric: ≥15 years.

### 2.3. Histopathological Evaluation

A total of 3 µm sections were stained with hematoxylin and eosin (HE), with and without bleaching, for re-examination by two independent pathologists (IP, JP). The bleaching was made by incubation in 0.25% potassium permanganate for 1 h, followed by incubation in 2% oxalic acid for a maximum of 10 min (depending on the amount of pigment). Tumors were divided according to the histological classification into benign melanoma and malignant melanoma. The histological features used to do this classification were: tumor vascular emboli (present, absent), nuclear grade (I—when nuclei had minimal variations in shape and size compared to normal nuclei; II—moderate alterations on nuclear shape; III—irregular and larger than normal nuclei), nucleolar size (small, medium, large), and mitotic count (more than 10 mitosis per ten high power fields (HPF)). Tumors presenting low mitotic count could carry other characteristics of malignancy. If a benign or malignant classification could not be reliably applied to a particular tumor this would be removed from the study. Tumors that did not resist bleaching were also eliminated.

The following histopathological features considered by [25,26] were evaluated; presence of epidermal ulceration (absent and present); circumscription (absent and present); degree of pigmentation (absent, slight, medium, high, and very high) and cell shape (epithelioid, spindle, and mixed).

### 2.4. Immunohistochemistry

For immunohistochemistry (IHQ), 3 µm sections were mounted on silane-coated slides. Immunolabeling was carried out with a commercial detection system (NovoLink Polymer Detection System; Novocastra, Leica Biosystems, Newcastle, UK) according to the manufacturer’s instructions. Briefly, tissue sections were dewaxed in xylene for 15 min and hydrated through a decreasing series of alcohol concentrations, 5 min on each, ending in tap water. Antigen-retrieval was made by microwave treatment in a solution of citrate buffer (0.01 M pH 6.0 ± 2) with 3 cycles of 5 min each at 750 W. After cooling the slides at room temperature for approximately 20 min, the already mentioned bleaching protocol was performed. Slides were passed in distilled water, dried and outlined with a hydrophobic pen (NovoPen, Leica, Newcastle, UK), and washed in phosphate buffered saline (PBS) solution (pH = 7.4) for 5 min. Endogenous peroxidase blocking was made through incubation with 3% hydrogen peroxide for 5 min, the slides were washed in PBS for 5 min, and endogenous protein blocking was performed for 5 min. After blocking non-specific binding, primary antibody E-cadherin/CDH1 Antibody (4A2C7, Invitrogen, Waltham, MA, USA) diluted 1:50 in PBS was incubated in a humidified chamber at 4 °C overnight. Slides were washed with PBS for 10 min and incubated with secondary antibody. Immunolabeling was visualized by incubation with 3,3′—diaminobenzidine tetrahydrochloride (DAB) chromogen. After washing in distilled water, slides were dehydrated and counterstained with Gill’s hematoxylin and cover slipped.

### 2.5. Immunohistochemical Evaluation

E-cadherin immunolabeling was evaluated blindly and semi-quantitatively by two independent pathologists (IP, JP), determining the extent of labeling (percentage of positive cells), the labeling intensity and labeling localization. Positivity was indicated by a brown membranous and/or cytoplasmatic labeling. Internal positive control (epidermal staining) and negative control (omission of primary antibody) were included in each staining run [27].

Regarding labeling extension, it was scored as 0—negative, 1—<25% labeled cells; 2—25–50% labeled cells and 3—>50% labeled cells. Intensity was scored as: 0—negative, 1—weak, 2—moderate, and 3—strong [27].

### 2.6. Statistical Analysis

Qui-square (X^2^) test of independence and Fisher exact test were used to determine whether E-cadherin labeling differed by histological classification (benign or malignant) and to evaluate any correlation with other histological features studied. Mann–Whitney was used to compare medians of labeling extension and intensity between groups. All the results were considered significant when *p* < 0.05. The analyses were performed using Jamovi (version 2.3.2) statistical software.

## 3. Results

### 3.1. Clinical Information

The study group included 60 horses, although clinical reports were not available for all. Regarding gender, 28 were males and 28 females, with an average age of 14.2 ± 5.39 years, the youngest being 2 years old and the oldest 26 years old. A total of 3 horses were young (≤5 years old), 13 were adults (between 6 and 14 years old), and 40 were geriatric (≥15 years old). The most common coat color was grey (*n* = 47) followed by cremello (*n* = 2), buckskin (*n* = 1), and brown (*n* = 1). Affected breeds were Pure-breed Lusitano (*n* = 27), Crossbreed (*n* = 19), Arabian (*n* = 3), and Warmblood (*n* = 2). The tumoral masses were distributed along the perianal region (*n* = 27), tail (*n* = 24), lips (*n* = 2), proximal limb (*n* = 2), trunk (*n* = 1), vulva (*n* = 1), neck (*n* = 1), and parotid gland (*n* = 1).

### 3.2. Histopathologic Results

A total of 72 melanocytic tumors were classified according to the histological features mentioned above as benign (*n* = 31) and malignant melanomas (*n* = 41).

Regarding histological classification (benign versus malignant) and histological features, there was no association between the classification and degree of pigmentation (*p* = 0.265), ulceration (*p* = 0.461), cell shape (*p* = 0.261), or circumscription (*p* = 0.439). Although scarce and difficult to identify, mitotic figures were only expressed in malignant tumors. The distribution of histological features used to classify tumors as benign and malignant is presented in Table 1.

Any association between histological classification and clinical features was found, namely gender (*p* = 0.189), age (*p* = 0.184), breed (*p* = 0.930), mass localization (*p* = 0.2), and coat color (*p* = 0.361). However, all the tumors belonging to horses with non-grey coat colors were malignant, presenting all the histological features previously described with association to malignancy: vascular emboli, >10 mitosis per 10HPF, nuclear grade 2 to 3, and medium to large nucleolar size. Furthermore, for the most part, malignant tumors belong to geriatric (≥15 years old) or adult horses who are 10 or more years old (*n* = 32). Two of the horses included in the study presented more than one tumor mass; however, no differences in the histological classification were observed between tumors of the same horse. One of the horses had nine malignant melanomas and another had five benign melanomas.

### 3.3. Immunohistochemical Results

**Figure 1:**
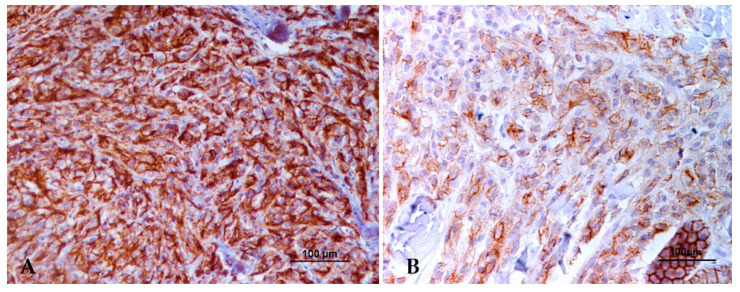
Strong E-cadherin immunolabeling, with more than 50% of labeled cells (**A**) and with 25–50% of labeled cells (**B**). In both images is visible a membranous and cytoplasmatic labeling.

Regarding the E-cadherin extent of labeling, 50 of 72 tumors (69.4%) presented more than 50% of labeled cells corresponding this percentage to 29/41 (70.7%) malignant melanomas and 21/31 (67.7%) benign melanomas; 13/72 tumors (18.1%) presented between 25–50% of labeled cells corresponding to 6/41 malignant melanomas (14.6%), and 7/31 benign melanomas (22.6%); 6 tumors presented <25% of labeled cells corresponding to 3/41 malignant melanomas (7.3%) and 3/31 benign melanomas (9.7%) and only 3/41 malignant melanomas (7.3%) did not present E-cadherin immunolabeling. No significant statistical differences were noted for the E-cadherin extent of labeling between benign and malignant melanomas (*p* = 0.388) with both presenting high percentages of labeled cells. Figure 1 represents different extensions of labeling in two different malignant melanomas.

**Figure 2:**
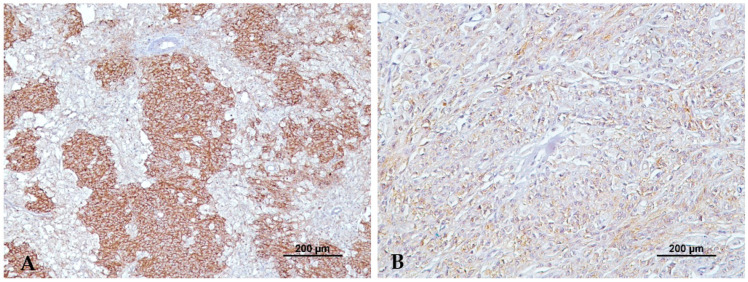
Diffuse E-cadherin immunolabeling with strong intensity (**A**) and low intensity (**B**).

Regarding the E-cadherin intensity of labeling, 23/72 tumors (31.9%) presented strong intensity (12/41 (29.3%) malignant melanomas and 11/31 (35.5%) benign melanomas), 31/72 tumors (43.1%) presented moderate intensity (17/41 (41.5%) malignant melanomas and 14/31 (45.2%) benign melanomas), and 15/72 tumors (20.8%) presented low intensity (9/41 (22%) malignant melanomas and 6/31 (19.4%) benign melanomas). No significant statistical differences were observed for the E-cadherin intensity of labeling (*p* = 0.458) between malignant and benign melanomas. Figure 2 represents different intensities of labeling in two different malignant melanomas.

Regarding E-cadherin labeling localization, 16/72 tumors (22.2%) had a membranous distribution (9/41 (22%) malignant melanomas and 7/31 (22.6%) benign melanomas), 35/72 tumors (48.6%) had a cytoplasmatic labeling (18/41 (43.9%) malignant melanomas and 17/31 (54.8%) benign melanomas), and mix distribution in 19/72 (26.4%) tumors (12/41 (29.3%) malignant melanomas and 7/31 (22.6%) benign melanomas). There was no statistical association between histological classification and E-cadherin labeling localization (*p* = 0.688).

Correlation between E-cadherin extension and histological features was not found; however, tumors presenting histological features of malignancy remained with a high extension of E-cadherin labeled cells (between scores 2 and 3). E-cadherin intensity and localization were not statistically associated with any of the histological features. Furthermore E-cadherin extension, intensity and localization were not associated with any clinical feature.

Although E-cadherin labeling extension mean of benign melanomas (2.55 ± 0.723) was greater than malignant melanomas (2.49 ± 0.925), there were no statistically significant differences between them (*p* = 0.927).

## 4. Discussion

In human medicine, Epithelial-Mesenchymal Transition has been an intense field of research in epithelial and non-epithelial tumors, including melanocytic tumors. The results obtained from EMT research explain in part the biology of melanocytic tumors and can be used as prognostic factors, leading to some effective therapeutical approaches [28,29,30]. However, the studies regarding EMT (including the role of E-cadherin) in veterinary medicine are scarce focusing mainly on small animals [20,31,32,33]. Thus, the molecular features of melanocytic tumors initiation, progression and pattern of invasion remain largely unknown in veterinary species. Although controversial, several authors mentioned that the downregulation of E-cadherin is a hallmark of EMT, being one of the most well-studied adhesion molecules in cancer research [34]. For some tumors, including melanomas, the loss of E-cadherin is referred to as being one of the factors that participate on the initiation of invasion and metastatic dissemination [34]. According to the author’s knowledge, studies about E-cadherin are not exist yet for equine melanocytic tumors.

The present study showed a non-expected immunolabeling pattern of E-cadherin in equine melanocytic tumors with some intriguing results that represent a contradiction to the studies made in other species. Regarding extension of labeling, 70.7% of melanomas presented more than 50% of labeled cells; 14.6% presented between 25–50%, and only three melanomas did not present any E-cadherin immunolabeling. A big difference is highlighted when compared to other studies. Veloso et al., 2020 reported that from the 38 canine cutaneous melanomas analyzed and 42% did not present any expression of E-cadherin; 5% presented 25–50% of labeled cells and 24% presented more than 50% of labeled cells. Furthermore, in the same study, 20 oral melanomas were analyzed with 55% of tumors not presenting any E-cadherin expression, 10% with 25–50% of labeled cells, and 15% with more than 50% of labeled cells. Although these results contrast sharply with ours, they correlate well with the clinicopathological behavior of melanocytic tumors in dogs since they are considered more aggressive, invasive, and present high metastatic rates [1].

In contrast to small animal and human literature, in the present study, there was not statistically significant differences in E-cadherin immunolabeling between benign and malignant equine melanomas, with both presenting an overall high immunolabeling of E-cadherin. Silvestri et al., 2020 made a comparative evaluation of E-cadherin expression between cutaneous benign and malignant melanomas. The expression was associated with histological diagnosis, that is, the percentage of positive cells was significantly higher in the benign than in malignant melanomas. Downregulation of E-cadherin in canine oral melanoma was also reported by Pisamai et al., 2017, which correlates once more the loss of this adhesion molecule with the aggressive behavior of these tumors.

Apparently, these differences in E-cadherin expression between equine melanocytic tumors and other species seem to make sense when comparing the biological behavior. Over the years, equine melanocytic tumors have intriguing clinicians and pathologists. These tumors present a benign clinical behavior being less invasive and presenting lower metastatic rates even in the presence of histological features of malignancy. The most common complication of these tumors is the direct effect of mass growth [5,26,35,36]. The results of our study seem to indicate that equine melanomas maintain a high E-cadherin expression for a longer time which is a possible justification for the less invasiveness of these tumors. Furthermore, looking at the genetic factors of tumor initiation in horses, it seems to make sense that there is high E-cadherin expression and simultaneously an overgrowth of the tumor mass. These tumors are more common in grey horses which have an agouti signaling protein (ASIP) loss of function mutation that leads to an increase of melanocortin-1 receptor (MC1R), resulting in higher levels of MITF [7,8,37,38]. MITF is the prominent regulator factor of EMT-like process in melanocytic tumors [18]. According to MITF expression levels, tumor cells can acquire two distinct phenotypical states: one state with MITF ^HIGH^ cells that is characterized by a proliferative phenotype with high E-cadherin levels and lower invasion, which drives melanoma tumor growth; the other is a MITF ^LOW^ cells characterized by invasive behavior with low E-cadherin expression. Several studies showed a positive correlation between MITF and E-cadherin, with MITF expression leading to high expression of E-cadherin. Given the tremendous plasticity and heterogenicity of melanocytic tumor cells, both states could be present within the same tumor [18,39,40,41,42,43].

The fact that some malignant tumors with more than 50% of E-cadherin labeled cells also presented tumor vascular emboli could be related to the presence in the same tumor of cells with MITF ^HIGH^ state and cells with MITF ^LOW^ state, with the last being capable of invading the ECM reaching the vessels [43]. Furthermore, it has recently been reported an alternative method of intravasation called “*passive intravasation*” in which tumor cells reach the vessels and possibly metastasize through passive shedding [16,17,44]. Passive intravasation focuses on cells that do not undergo EMT-like changes. These cells can reach the vessels because they got attached to EMT cells. Another possibility is that, during tumor progression, several changes occur in the ECM and tumor microenvironment that create the possibility of these shedding cells to pass through this weakened environment until reaching the vessels. Studies propose that both active and passive intravasation can simultaneously occur, possibly justifying the intravascular cells found in samples with high levels of E-cadherin [16,17,44]. Nevertheless, the high levels of MITF and E-cadherin make the number of cells able to invade the ECM much smaller, which possibly justify the lower prevalence of metastasis in horses.

Regarding E-cadherin localization, the absence of association with histological classification is in accordance with some articles and in disagreement with others. Hodorogea et al., 2019 report that the transition from membranous to cytoplasmatic labeling is related to the malignant transformation of tumors. Silvestri et al., 2020 reported no differences between E-cadherin localization and histological classification since most tumors presented both membranous and cytoplasmatic labeling. Our study showed that a great part of tumors presented a cytoplasmatic (17 benign and 18 malignant melanomas) or mixed (7 benign and 12 malignant melanomas) localization. Maybe it could represent some degree of E-cadherin dysfunction, according to some authors.

The fact that some benign melanomas (*n* = 3) presented a low E-cadherin expression could be a possible indication that these tumors were passing through a period of transition to malignancy. Although it is not possible, it would be interesting to do follow-up studies and see if these benign melanomas would evolve into more invasive malignant forms in the future. As mentioned by Campagne et al., 2012 and MacKay 2019, benign melanomas in horses seem to be a premalignant state since these tumors have the potential to acquire histological features of malignancy at any time.

E-cadherin was not correlated with any of the histological features analyzed, which disagrees with some previous studies. Some authors suggested that E-cadherin expression was greater in epithelioid cells than in spindle cells [43,45]. This was not verified in our study maybe because most equine melanocytic tumors are composed of mixed cell shapes as mentioned by [46]. Silvestri et al., 2020 showed an association of E-cadherin expression with pigmentation referring that, tumors with more than 50% of pigmented cells tend to express more E-cadherin and amelanotic tumors tend to express less E-cadherin and are related with more aggressive behavior. Those studies agreed with some authors that propose pigmentation as a prognostic factor for canine melanoma [47]. However, our results did not reach the same conclusion, maybe because of the low number of amelanotic or low pigmented tumors in our sample. Amelanotic melanomas are rarely seen in horses, although they have been associated with more aggressive behavior [35]. Furthermore, according to the authors experience, the overall pigmentation of equine melanocytic tumors in horses seems to be higher compared with dogs.

All the literature regarding the benign behavior of equine melanocytic tumors refers to grey horses. In fact, this is not the reality when we look at non-grey horses. When these horses develop a melanocytic tumor, it tends to be much more aggressive with faster progression to malignancy, higher invasiveness, and higher metastatic rates [5,7,35,48]. These facts lead researchers to think that solid colors horses have different factors involved in melanocytic tumor initiation and progression compared with grey horses. The tumors in our sample that came from solid-color horses presented every histological feature of malignancy that were evaluated. Furthermore, some of these tumors presented only moderated extensions of E-cadherin labeling (25–50%) with low to moderate intensity of labeling. Combining these facts, they are prone to become more invasive. Although we do not have clinical information about presence or absence of metastasis in these horses, which would be interesting to study, the overall histological and immunohistochemical evaluation of these tumors in solid-color horses seems to match with the clinical behavior described in the literature.

In the present study, regarding clinical information, most part of the horses were grey (*n* = 47) and only a few were non-grey horses (*n* = 4). This result is in accordance with the literature since the genetic trait that confers the greying phenotype (4.6k duplication on intron 6 of STX_17_ gene) is the initiating factor for melanoma development [7,8,37]. Most horses were geriatric (*n* = 40) with only a few young horses which agree with the literature, since melanocytic tumors development is age dependent, beginning approximately between six and seven years old when the coat color starts greying [35,49,50]. Furthermore, most part of melanomas were carried by older grey horses. Once more the literature corroborates this result. Malignancy is a time-related feature of equine melanocytic tumors and contrary to other species it can take years to occur, being more common after geriatric age is achieved [35]. There is a high likelihood that every geriatric grey horse ends up developing melanoma [35]. However, this work could not demonstrate a statistically significant correlation between malignancy and age, probably due to the absence of clinical information in a great part of the cases, which impacts the statistics.

The absence of a statistical correlation between gender and histological classification is in accordance with previous studies in dogs [3,51]. The breed predisposition is still a subject of discussion since it is thought that certain breeds (Lipizzaner, Andalusian, and Lusitano) may have a higher prevalence of these tumors because they have a higher number of grey horses and not because there is a true breed predisposition [7,36]. The tumoral mass localization is in accordance with the most common anatomical places referred on the literature for these tumors. No association was seen between localization and histological classification, as mentioned in equine literature, contrary to dogs where tumor localization has massive importance, with oral melanomas being almost all malignant [3,35].

Histological classification of equine melanocytic tumors is a technical question of great debate among pathologists [26]. The histological criteria created for the evaluation of small animal melanocytic tumors and does not seem to apply to horses since it does not correlate with the typical malignant behavior that is expected when we compare it with some types of canine melanoma [35]. Authors suggest that different molecular patterns of equine melanocytic tumors, such as the high E-cadherin expression in malignant tumors, could be a possible explanation for the absence of correlation between histological classification and clinical behavior, as was mentioned above. Nevertheless, nuclear grade, nucleolar size, tumor vascular emboli, and mitotic count seem to be good evaluation parameters as mentioned by [26]. The overall low mitotic count in most of the tumors presented in this study is contradictory regarding tumor clinical behavior and it could be a possible mistake originating from the limitations of routine hematoxylin-eosin evaluation [52]. The mass expansion, typical of these tumors, needs to be correlated with a proliferative profile. However, low mitotic counts are reported by several authors during hematoxylin-eosin evaluation of equine melanocytic tumors, being, therefore, considered a common pitfall. As so, the mitotic count could be underestimated on these tumors [26,46]. The pigmentation was not related to histological diagnosis either. Both equine benign and malignant melanomas are characterized as being highly pigmented tumors. Amelanotic melanoma is rarely identified in horses and being considered clinically more aggressive by some authors [35]. There are no studies analyzing the correlation between tumor pigmentation and clinical behavior in horses. Studies in dogs have contradictory results regarding pigmentation not yet considering it as a reliable factor, neither for malignancy nor clinical outcome, since there are no validated cutoff points [3,22,47]. None of the cell types evaluated were associated with malignancy, with tumors presenting a mixed cell shape dispersed heterogeneously along the mass. This is a common feature in equine melanocytic tumors since most of the tumors present various cellular shapes [46].

One of the main limitations of this study is that there was no follow-up of these cases, so we do not know if the horses in the long term developed metastases or if the tumors acquired a more invasive character. Therefore, it is not possible to obtain a correlation between the E-cadherin immunolabelling and the metastasis/invasiveness occurrence. It is not possible to gauge the true biological value of this marker in equine melanomas. However, this study remains a starting point to better understand the biology of equine melanomas and what potential molecular differences exist that may account for the differences in the clinical behavior of these tumors in horses.

## 5. Conclusions

The results of our study showed a significantly different immunostaining pattern of E-cadherin on equine melanocytic tumors. It appears that these tumors remain with high immunolabeling of E-cadherin over time, even in the presence of malignant histologic features, which may be one of the factors that allow these tumors to develop over the years without becoming clinically invasive and presenting a lower prevalence of metastases. This evidence correlates quite well with the known benign clinical behavior that these tumors have in horses.

## Figures and Tables

**Table 1:** Distribution of histological features statistically associated with histological classification.

Histological Classification	Nuclear Grade			Nucleolar Size			Mitotic Count			Tumor Vascular Emboli	
	I	II	III	Small	Medium	Large	0	<10	>10	Absent	Present
Benign	26	5	0	28	3	0	31	0	0	31	0
Malignant	0	10	31	9	29	3	28	3	10	13	28

## Data Availability

No new data were created.
